# RSK-mediated down-regulation of PDCD4 is required for proliferation, survival, and migration in a model of triple-negative breast cancer

**DOI:** 10.18632/oncotarget.8375

**Published:** 2016-03-25

**Authors:** Rafael Cuesta, Marina K. Holz

**Affiliations:** ^1^ Department of Biology, Stern College for Women of Yeshiva University, New York, New York 10016, USA; ^2^ Department of Molecular Pharmacology, Albert Einstein College of Medicine, Bronx, New York 10461, USA; ^3^ Albert Einstein Cancer Center, Albert Einstein College of Medicine, Bronx, New York 10461, USA

**Keywords:** RSKs, PDCD4, translational control, MAPK upregulation, TNBC biology

## Abstract

The p90 ribosomal S6 kinase (RSK) is a family of MAPK-activated serine/threonine kinases (RSK1-4) whose expression and/or activity are deregulated in several cancers, including breast cancer. Up-regulation of RSKs promotes cellular processes that drive tumorigenesis in Triple Negative Breast Cancer (TNBC) cells. Although RSKs regulate protein synthesis in certain cell types, the role of RSK-mediated translational control in oncogenic progression has yet to be evaluated. We demonstrate that proliferation and migration of TNBC MDA-MB-231 cells, unlike ER/PR-positive MCF7 cells, rely on RSK activity. We show that RSKs regulate the activities of the translation initiation factor eIF4B and the translational repressor PDCD4 in TNBC cells with up-regulated MAPK pathway, but not in breast cancer cells with hyperactivated PI3K/Akt/mTORC1 pathway. These results identify PDCD4 as a novel RSK substrate. We demonstrate that RSK-mediated phosphorylation of PDCD4 at S76 promotes PDCD4 degradation. Low PDCD4 levels reduce PDCD4 inhibitory effect on the translation initiation factor eIF4A, which increases translation of “eIF4A sensitive” mRNAs encoding factors involved in cell cycle progression, survival, and migration. Consequently, low levels of PDCD4 favor proliferation and migration of MDA-MB-231 cells. These results support the therapeutic use of RSK inhibitors for treatment of TNBC with deregulated MAPK/RSK pathway.

## INTRODUCTION

The p90 ribosomal S6 kinase (RSK) comprises a family of four serine/threonine kinases in humans (RSK1-4) that are controlled by the MAPK/ERK pathway. The four family members share 73% to 80% sequence homology. Although the high degree of homology suggests some functional redundancy, evidence supports the existence of isoform-specific functions [1, 2]. RSKs regulate cellular growth and proliferation, cell-cycle progression, survival, and migration. Therefore, up-regulation of RSKs contributes to tumor development and progression [1–3].

Hyperactivation of RSK signaling is found in many cancers, including breast cancer [1]. All four RSKs are expressed at different levels in breast tumors and cancer cells (Human Protein Atlas; http://www.proteinatlas.org) [4]. Increased expression or activation of RSK1 and RSK2 are detected in human breast cancer tissues compared to normal breast tissues, particularly in TNBC tissues [5–7]. Inhibition and/or silencing of RSK1 and RSK2 reduce proliferation, survival, migration, and invasion of breast cancer cells, especially TNBC cells, and prevent breast cancer stem cell growth, underscoring their role in breast tumorigenesis [6–13]. The role of RSK3 and RSK4 in breast cancer biology is controversial. RSK4 shows anti-tumorigenic activity when over-expressed in TNBC MDA-MB-231 cells [14]. However, over-expression of RSK3 or RSK4 promotes proliferation of ER/PR-positive MCF7 breast cancer cells upon inhibition of PI3K/mTORC1 pathway and promotes resistance to PI3K inhibitor in a xenograft model [5].

RSKs control oncogenic processes through the regulation of transcription factors and/or cellular modulators [1–3, 7, 11, 12]. RSKs regulate protein synthesis, and therefore, RSK-mediated translational control may also play a critical role in the regulation of tumorigenic cellular events, but this mechanism has not been investigated in depth. RSKs phosphorylate the translation initiation factor eIF4B and the ribosomal protein S6, which results in increased cap-dependent translation [15, 16]. The activation of eIF4B enhances the RNA helicase activity of eIF4A in unwinding secondary structures in the 5′ untranslated region (5′-UTR) of mRNAs [17, 18]. eIF4A is a component of the eIF4F complex, which also includes the mRNA 5′ cap binding protein eIF4E and eIF4G, a large scaffolding protein upon which the translation apparatus assembles. eIF4F recruits mRNA to the small ribosome subunit, facilitating the scanning of the 5′-UTR and the assembly of the 80S ribosome-initiation complex at the AUG start codon [19]. In addition to the ERK/RSK pathway, the PI3K/Akt/mTORC1 pathway regulates the activity of eIF4F through phosphorylation of eIF4E binding proteins (4E-BPs) and the p70 ribosomal S6 kinase (S6K) [20]. Phosphorylation of the translational repressors 4E-BPs prevents their binding to eIF4E, which allows the assembly of eIF4F complex and stimulates cap-dependent mRNA translation [21, 22]. Concurrently, phosphorylation of S6K results in the activation of eIF4B and the degradation of the tumor suppressor Programmed Cell Death 4 (PDCD4), a negative regulator of protein synthesis [17, 23]. These two events enhance eIF4A activity, and favor the translation of specific mRNAs, as described above [17, 23, 24]. In addition to the regulation of eIF4A, RSKs may also regulate eIF4F through the control of mTORC1 activity in certain cell lines [25–27]. Deregulation of eIF4F is observed in many cancers, and results in increased translation of specific mRNAs that encode for proteins involved in the regulation of cellular growth and proliferation, enhanced survival, migration, and invasion [28, 29]. These results suggest that increased eIF4F activity as a consequence of RSK up-regulation may play a relevant role in the control of cellular processes that drive cancer development and progression. Conversely, inhibition of RSKs or silencing of RSK1 and RSK2 in melanoma cells results in reduced mTORC1 activity and therefore, in decreased eIF4F activity, which correlates with reduced tumor growth in mice [25]. However, the contribution of mTORC1-independent RSK regulation of mRNA translation to cellular processes that drive tumorigenesis has yet to be evaluated.

In this study, we compared the role of RSKs in the control of protein synthesis in several breast cancer cell lines. Interestingly, we identified PDCD4 as a RSK substrate. The phosphorylation-induced degradation of PDCD4 and the phosphorylation of eIF4B relied on RSK activity in TNBC cells with activated MAPK signaling (MDA-MB-231 and MDA-MB-436), but not in the cells in which the PI3K pathway is the main oncogenic driver (MDA-MB-468, MCF7, and T47D) [30]. Moreover, we observed that RSK1 and RSK2 isoforms regulated PDCD4 protein levels, which was required for the translation of mRNAs encoding factors involved in cell cycle progression and survival, such as Cyclin D1 and Bcl2. Reduced PDCD4 translational repression activity was important for proliferation, survival and migration of MDA-MB-231 cells. Our findings support the potential value of RSK or RSK-regulated translation factors as targets for the treatment of TNBC tumors with up-regulated MAPK signaling.

## RESULTS

### RSKs control proliferation, survival, and migration of TNBC MDA-MB-231 cells

RSKs control proliferation, survival, migration, and invasion of breast cancer cells, particularly of TNBC cells [6–10]. To assess the contribution of RSK to these cellular processes, we selected two cell lines: MCF7 as a model for ER/PR-positive breast cancer subtype and MDA-MB-231 as a model for the TNBC subtype. Additionally, by using these cell lines, we could compare the role of RSKs in cells harboring activating mutations in the PI3K/Akt/mTORC1 pathway such as MCF7, with their role in cells harboring a constitutively activated MAPK pathway such as MDA-MB-231 [30].

**Figure 1:**
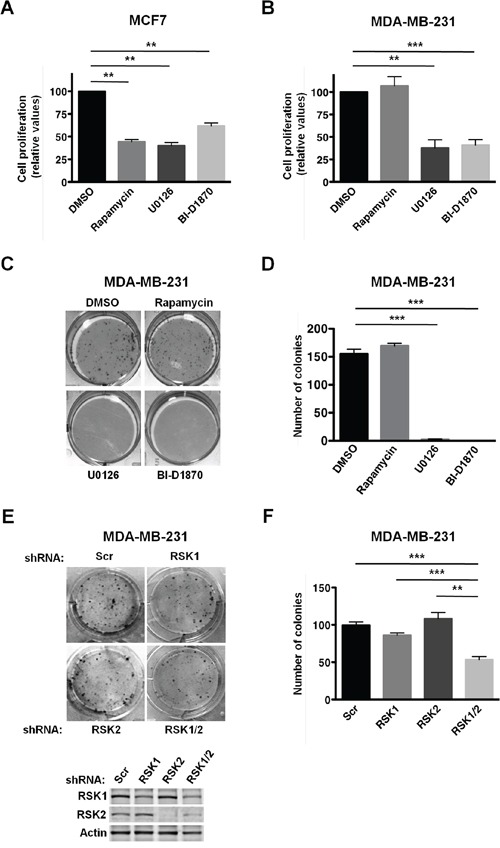
PMA-stimulated proliferation of MDA-MB-231 cells relies on RSK activity **A.** MCF7 cells were grown in 0.5% FBS media with PMA (50 ng/ml) and vehicle (DMSO), rapamycin (20 nM), U0126 (10 μM), or BI-D1870 (10 μM) for 3 days. Media were replaced with fresh media containing PMA and vehicle or inhibitors daily. Viable cells were estimated by neutral red uptake assays, and values represented as mean percentage ± SEM relative to vehicle (DMSO) treated cells (100%) determined from three independent assays (*p<0.05; **p<0.01; ***p< 0.001). **B.** MDA-MB-231 cells were grown, and results were obtained and analyzed as described in A. **C.** MDA-MB-231 cells were grown in 10% FBS media supplemented with vehicle (DMSO), rapamycin (20 nM), U0126 (10 μM), or BI-D1870 (10 μM) for 14 days. Media were replaced with fresh media containing vehicle or inhibitors every three days. Cells were fixed with methanol and stained with crystal violet. **D.** Quantification of anchorage-dependent focus assays shown in C. Colonies with 50 or more cells were counted. Results from three independent assays represented as means ± SEM. (*p<0.05; **p<0.01; ***p<0.001). **E.** Control (Scr), RSK1-, RSK2-, and RSK1/2-silenced MDA-MB-231 cells were grown in 10% FBS for 14 days, and processed as described in C. Expression of RSK1 and RSK2 in the above-mentioned cell lines was determined by immunoblotting analysis. **F.** Quantification of the anchorage-dependent focus assays shown in E was performed as described in D.

First, we evaluated the requirement of RSKs for the proliferation of MCF7 and MDA-MB-231 cells. To this end, cells were treated with the phorbol ester PMA to stimulate RSK activity, and with either the vehicle (DMSO), or inhibitors of mTORC1 (rapamycin), MEK1/2 (U0126), or RSKs (BI-D1870) for 3 days. Inhibition of the MAPK/RSK pathway suppressed proliferation of both cell lines, although MCF7 cells were less sensitive to RSK inhibition (∼ 40%, Figure 1A) than MDA-MB-231 cells (∼ 60%, Figure 1A and 1B). In contrast, inhibition of the mTORC1 pathway only suppressed proliferation of MCF7 cells (Figure 1A and 1B), consistent with our previously published observations [31, 32]. The requirement of the MAPK/RSK pathway for MDA-MB-231 cells proliferation was further confirmed by anchorage-dependent clonogenic assays (Figure 1C and 1D). Notably, specific silencing of RSK1 and RSK2, the two oncogenic RSK isoforms, decreased the colony formation capability of MDA-MB-231 cells (Figure 1E and 1F). Single silencing of RSK1 or RSK2 did not significantly affect cellular proliferation, suggesting a functional redundancy of these two RSK isoforms in this cell line. Additionally, we observed that RSK1/2 silencing did not suppress proliferation as efficiently as the RSK inhibitor, which could be due to incomplete RSK1/2 silencing or a compensatory role of RSK3 and/or RSK4 in this cellular process. These results indicated that proliferation of MDA-MB-231 cells relies on the MAPK/RSK pathway, while proliferation of MCF7 cells depends on both PI3K/Akt/mTORC1 and MAPK/RSK pathways.

**Figure 2:**
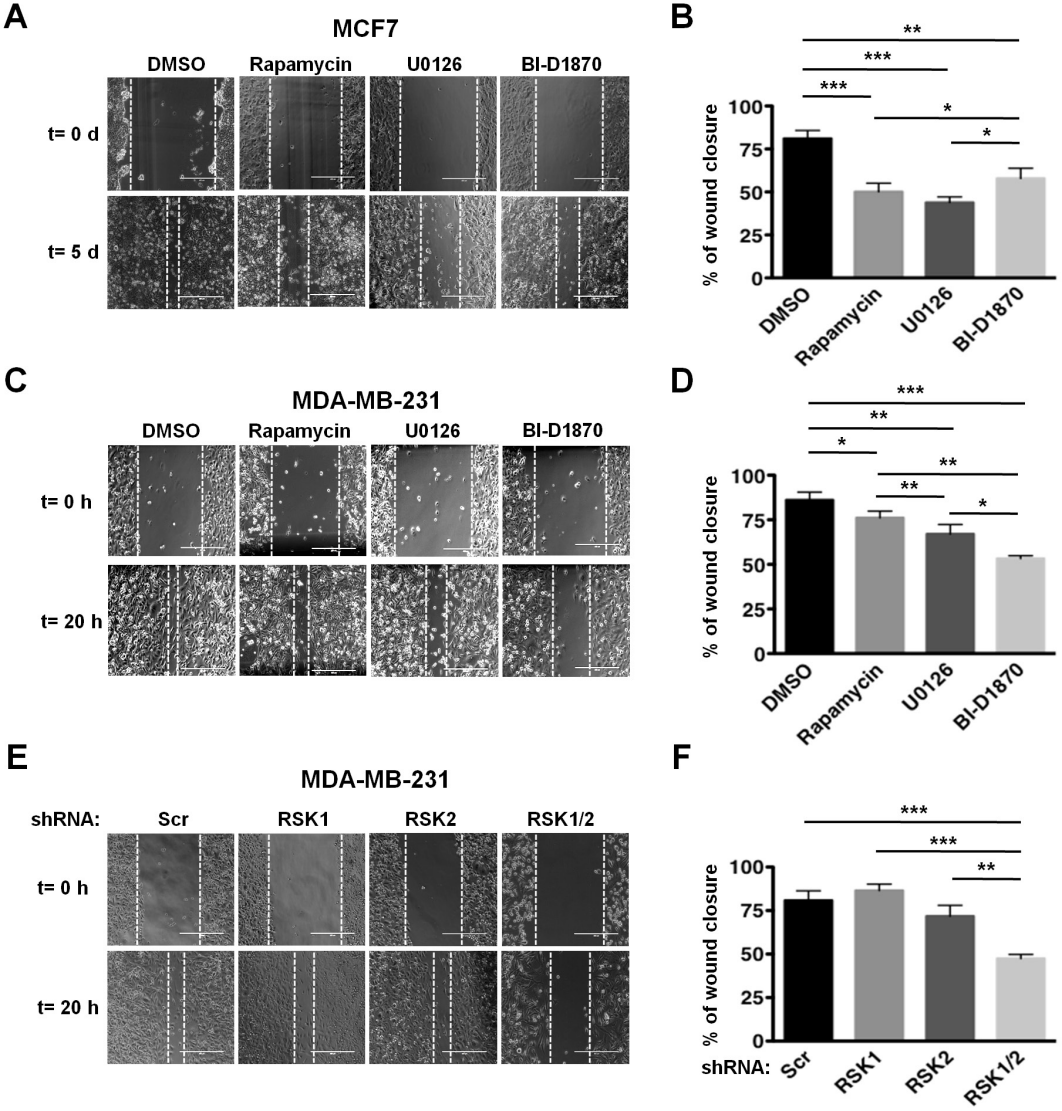
RSKs control migration of MDA-MB-231 cells **A.** MCF7 cells were subjected to wound healing assays in 1% FBS media containing vehicle (DMSO), rapamycin (20 nM), U0126 (10 μM), or BI-D1870 (10 μM). Media were replaced with fresh media containing vehicle or inhibitors daily. Representative photographs at the indicated times from three independent experiments performed in triplicate are shown. Magnification: x10. **B.** Percentage of wound recovery was determined in the experiments shown in A as described in Materials and Methods, and results were quantified as means ± SEM. (*p<0.05; **p<0.01; ***p<0.001). **C.** MDA-MB-231 cells were subjected to wound healing assays as described in A. **D.** Percentage of wound recovery was determined in the experiments shown in C, and results were quantified as in B. **E.** Control (Scr), RSK1-, RSK-2, or RSK1/2-silenced MDA-MB-231 cells were subjected to wound healing assays in 1% FBS media. **F.** Percentage of wound recovery was determined in the experiments shown in E, and results were quantified as in B.

Next, we evaluated the requirement of RSKs for cellular migration in both cell lines by wound healing assays. Using pharmacological inhibitors, we observed that while both PI3K/Akt/mTORC1 and MAPK/RSK pathways controlled MCF7 cells migration (Figure 2A and 2B), RSKs regulated MDA-MB-231 cells migration (Figure 2C and 2D). The requirement for RSKs for MDA-MB-231 cell migration was confirmed using RSK1/2-silenced cells (Figure 2E and 2F). While knockdown of each isoform individually did not affect migration due to compensatory rescue by the other isoform, the combined RSK1/2 suppression had an effect similar to that of the RSK inhibitor.

Collectively, these experiments demonstrate that the MAPK/RSK pathway is the main driver of cellular proliferation and migration of PMA-stimulated MDA-MB-231 cells.

### RSKs regulate phosphorylation of eIF4B and S6, and PDCD4 levels in TNBC cells with up-regulated MAPK pathway

**Figure 3:**
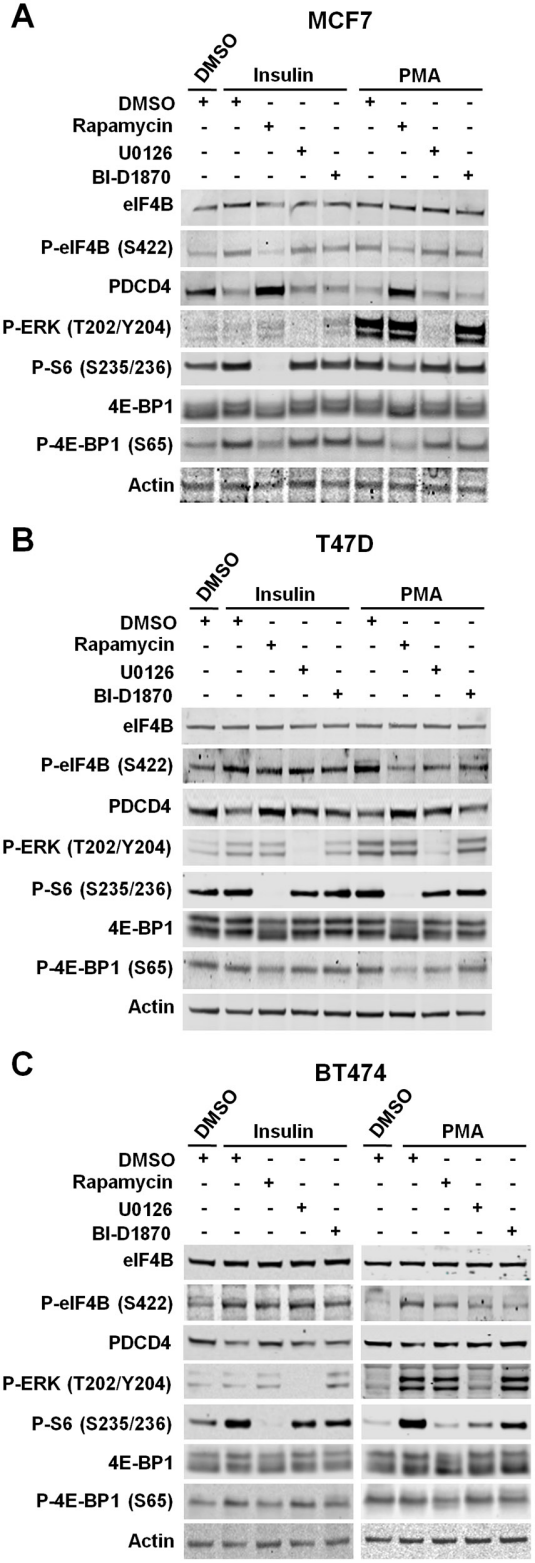
The PI3K/Akt/mTORC1 pathway controls eIF4B phosphorylation and PDCD4 levels in ER/PR-positive cells **A.** MCF7 cells were deprived of serum for 24 h, then treated with vehicle (DMSO), rapamycin (20 nM), U0126 (10 μM), or BI-D1870 (10 μM) for 30 min, followed by stimulation with insulin (100 nM) or PMA (50 ng/ml) for 2 h. Indicated proteins were analyzed by immunoblots. **B.** Serum-starved T47D cells were treated as described in A. Indicated proteins were analyzed by immunoblots. **C.** BT474 cells were deprived of serum for 24 h and then treated as described in A. Indicated proteins were analyzed by immunoblots.

**Figure 4:**
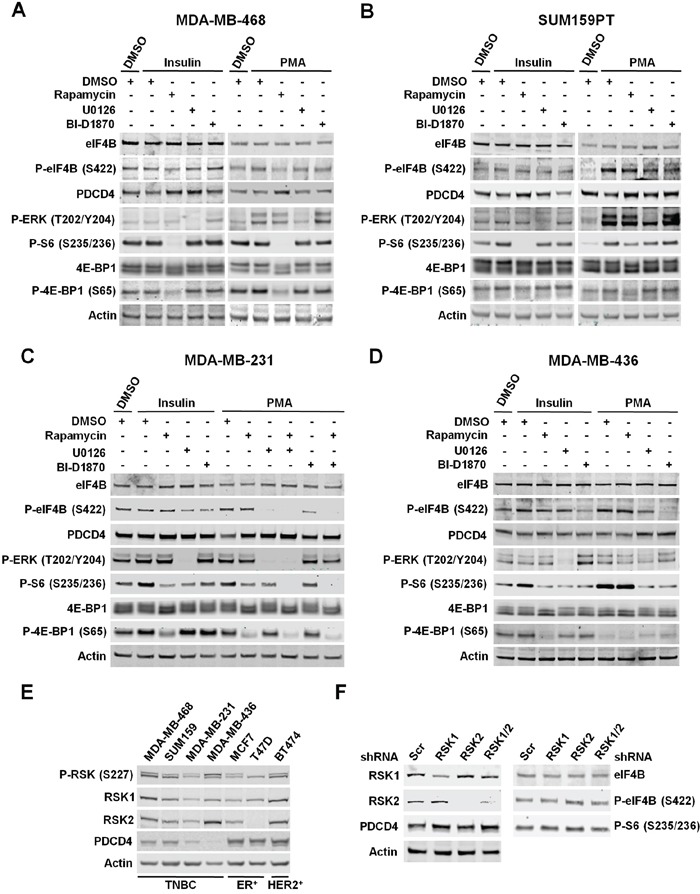
RSKs regulate phosphorylation of eIF4B and the levels of PDCD4 in TNBC cells with up-regulated MAPK pathway **A.** MDA-MB-468 cells were deprived of serum for 24 h, then treated with vehicle (DMSO), rapamycin (20 nM), U0126 (10 μM), or BI-D1870 (10 μM) for 30 min, followed by stimulation with insulin (100 nM) or PMA (50 ng/ml) for 2 h. Indicated proteins were analyzed by immunoblots. **B.** Serum-starved SUM159PT cells were treated as described in A. Indicated proteins were analyzed by immunoblots. **C.** MDA-MB-231 cells were deprived of serum for 24 h and then treated as described in A. Indicated proteins were analyzed by immunoblots. **D.** Serum-starved MDA-MB-436 cells were treated as described in A. Indicated proteins were analyzed by immunoblots. **E.** Cells were serum-starved for 24 h followed by stimulation with PMA for 2 h. Whole-cell extracts were resolved by SDS-PAGE and indicated proteins detected by immunoblot analysis. **F.** MDA-MB-231 cells were infected with lentiviruses expressing shRNAs targeted against a scrambled sequence (Scr), RSK1, RSK2, or RSK1/2. After selection, cells were serum-starved for 24 h followed by stimulation with PMA (50 ng/mL) for 4 h. Cell extracts were resolved by SDS-PAGE, and indicated proteins were analyzed by immunoblotting with specific antibodies.

To determine the role of RSKs in the regulation of protein synthesis, we used ER/PR-positive (MCF7 and T47D), ER/PR/HER2 positive (BT474), and TNBC cells (MDA-MD-468, MDA-MB-436, MDA-MB-231, and SUM159PT). Table 1 shows the oncogenic mutations in the PI3K/Akt/mTORC1 and/or MAPK pathways identified in these cell lines. We evaluated the phosphorylation status and total levels of mTORC1/S6K and/or RSK substrates in response to insulin stimulation, which primarily activates the PI3K/Akt/mTORC1 pathway, or PMA stimulation, which primarily activates the MAPK/RSK pathway. Phosphorylation of eIF4B at S422, S6 at S235/236, 4E-BP1 at S65 as well as the decrease in PDCD4 levels mostly depended on the PI3K/Akt/mTORC1 pathway, under any tested condition, in breast cancer cell lines in which only this pathway was up-regulated (MCF7, T47D, and MDA-MB-468) (Figure 3A, 3B and 4A). However, PI3K/Akt/mTORC1 dependency varies among these cell lines. Thus, T47D cells showed a lower effect of the mTORC1 pathway on the regulation of these proteins. These results could be explained by the reduced sensitivity of T47D cells to the mTORC1 inhibitor rapamycin, as determined by the partial inhibition of the well-characterized mTORC1 substrate 4E-BP1 in these cells compared to MCF7 and MDA-MB-468 cells. In addition, we observed a modest role of MAPK pathway in the regulation of eIF4B and S6 phosphorylation, and PDCD4 levels in T47D cells under both experimental conditions. Although MAPK pathway is also activated by PMA in MCF7 and MDA-MB-468 cells, we did not observe any effect of MAPK/RSK pathway on the regulation of these proteins. In MCF7 cells, this result could be explained by the strong PMA-induced activation of mTORC1 pathway [33]. Taken together, these results ruled out any significant effect of RSK-mediated translational control on the proliferation and migration of these cells lines.

**Table 1:** List of breast cancer cell lines used in this study

Cell line	ER	PR	HER2	Subtype	Gene amplification	Genetic alterations
**MCF7**	**+**	**+**	−	Luminal		*PIK3CA*, *CDKN2A*
**T47D**	**+**	**+**	−	Luminal		*PIK3CA*, *TP53*
**BT474**	**+**	**+**	**+**	Luminal		*TP53*
**MDA-MB-231**	−	−	−	Basal		*BRAF*, *CDKN2A*, *KRAS*, *NF2*, *TP53*, *PDGFRA*
**MDA-MB-436**	−	−	−	Basal		*BRCA1*, *TP53*
**MDA-MB-468**	−	−	−	Basal	EGFR	*PTEN*, *RB1*, *SMAD4*, *TP53*
**SUM159PT**	−	−	−	Basal		*PIK3CA, TP53, HRAS*

Sources: ER, PR and HER2 status and subtypes were taken from Neve et al. [54]; gene amplification and genetic alterations were taken from Lehmann et al. [46], Hu et al. [55] and Chaves et al. [56].

Remarkably, in TNBC cells with constitutively activated MAPK pathway, phosphorylation of eIF4B and S6, and PDCD4 levels were primarily regulated by MAPK/RSK activity upon PMA-stimulation (MDA-MB-231 and MDA-MB-436) (Figure 4C and 4D). In MDA-MB-231 cells, we also observed contribution of mTORC1 pathway to the regulation of S6 phosphorylation and PDCD4 levels under this experimental condition. Unlike the MDA-MB-436 cells, PMA stimulation partially activates mTORC1 in MDA-MB-231 cells, as determined by phosphorylation of the mTORC1-specific substrate 4E-BP1 at S65. Accordingly, insulin-induced activation of PI3K/Akt/mTORC1 pathway results in increased phosphorylation of S6, and a modest decrease in the levels of PDCD4 in both cell lines, while no effect was observed on phosphorylation of eIF4B (Figure 4C and 4D). These results indicated that agonist-activated MAPK/RSK pathway significantly contributes to the regulation of eIF4B and S6 phosphorylation, and PDCD4 levels in breast cancer cells with activating mutations in this pathway.

When breast cancer cells with either wild-type (BT474) or activating mutations in both PI3K/Akt/mTORC1 and MAPK pathways (SUM159PT) were analyzed, we observed that the regulation of eIF4B, S6 and PDCD4 mostly relies on the agonist-stimulated pathway. Hence, mTORC1 pathway played a more relevant role in insulin-stimulated cells, while MAPK pathway significantly contributed to the regulation of these proteins upon stimulation with PMA (Figure 3C and 4B).

Interestingly, we identified PDCD4 as a new substrate for RSKs in breast cancer cells, which agreed with recently published results in melanoma cells [34]. The levels of PDCD4 were much lower in TNBC cells, particularly in the cells with up-regulated MAPK pathway, compared to ER/PR-positive or ER/PR/HER2 positive cells upon PMA stimulation. These results suggested an important role of RSK-mediated PDCD4 down-regulation in the biology of TNBC (Figure 4E). Stimulation with PMA activated RSK1 and RSK2 at similar levels in all tested cell lines, as determined by Erk1/2-promoted phosphorylation of RSK1 at S221 and RSK2 at S227 by PDK1 (Figure 4E) [1]. These observations supported the contribution of PMA-induced activation of RSKs to the regulation of PDCD4 levels in these breast cancer cells, but not in cells with only the activating mutations in PI3K/Akt/mTORC1 pathway. In the latter, although activated, RSKs did not significantly contribute to PDCD4 regulation. The results shown in Figure 4E also suggest that PDCD4 is differentially regulated by other mechanisms in TNBC cells and ER/PR-positive or ER/PR/HER2-positive cells.

Using RSK1/2-silenced MDA-MB-231 cells, we confirmed the regulation of PDCD4 by RSK1 and RSK2 (Figure 4F). Surprisingly, we did not observe any effect of RSK1/2 depletion on the phosphorylation of eIF4B or S6, which suggested that RSK3 and/or RSK4 were implicated in these phosphorylation events (Figure 4F). Similarly, over-expression of RSK3 or RSK4 abrogated the dephosphorylation of eIF4B and S6 by PI3K, mTOR or dual PI3K/mTOR inhibitors in MCF7 cells [5]. These results identified isoform-specific functions of RSKs in control of protein synthesis, which could partially explain the incomplete suppression of cellular proliferation by RSK1/2 silencing compared to RSK inhibition, as proposed above (Figure 1D and 1F). Taken together, these results suggest a role of RSK-mediated translational control in the proliferation and migration of TNBC cells with up-regulated MAPK signaling.

### RSKs phosphorylate PDCD4 and promote PDCD4 protein degradation in MDA-MB-231 cells

**Figure 5:**
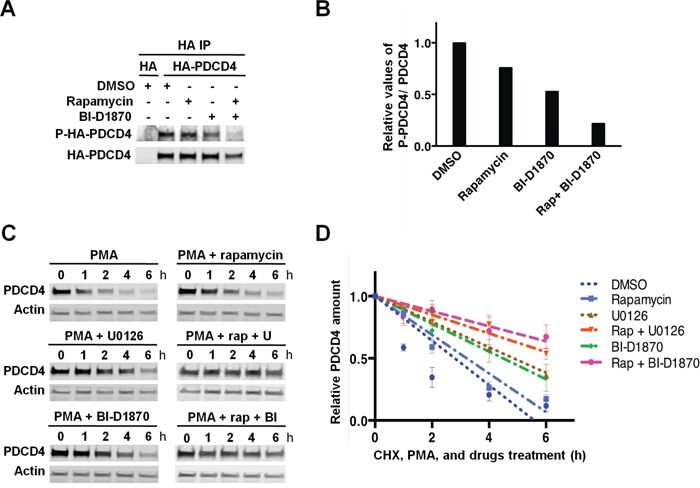
RSK-mediated phosphorylation of PDCD4 regulates PDCD4 protein stability in PMA-stimulated MDA-MB-231 cells **A.** MDA-MB-231 cells were transfected with an empty vector (HA) or a construct expressing HA-tagged PDCD4. Cells were serum-starved for 24 h, then treated with vehicle (DMSO), rapamycin (20 nM), and/or BI-D1870 (10 μM) for 1 h, followed by stimulation with PMA (50 ng/ml) for 30 min. HA-tagged PDCD4 protein was immunoprecipitated with anti-HA antibody, and phosphorylation was analyzed with an anti-RXRXXpS/T motif antibody. **B.** Phospho-HA-PDCD4 and HA-PDCD4 levels were quantified in the immunoblot shown in A using Image J software. Phospho-PDCD4 values were normalized by total PDCD4 values, and data were plotted relative to PMA-stimulated control cells (set to 1). **C.** Serum-starved MDA-MB-231 cells were treated with vehicle (DMSO), rapamycin (20 nM), U0126 (10 μM), or BI-D1870 (10 μM), as indicated, for 30 min before adding cycloheximide (CHX; 100 μg/mL) and PMA (50 ng/ml). Cell extracts were prepared at the indicated time points, resolved by SDS-PAGE, and PDCD4 and actin levels analyzed by immunoblotting. **D.** PDCD4 and actin levels were quantified using Image J software in immunoblots from three independent experiments performed as shown in C. PDCD4 values were normalized by actin values. Relative PDCD4 values to respective t=0 controls (set to 1) were represented as means ± SEM, and the best-fit linear regression curves were calculated using Graphpad Prism 6.

Our results indicated that the levels of the translational inhibitor PDCD4 are regulated by RSKs and the mTORC1 pathway in TNBC cells with hyperactivated MAPK pathway. Indeed, both Akt and S6K1, and RSKs have been shown to regulate the half-life of PDCD4 protein by phosphorylation at S67 and S457, and at S76 and S457, respectively [23, 34–36]. Therefore, we first investigated the phosphorylation of PDCD4 in MDA-MB-231 cells. Cells expressing HA-tagged PDCD4 were treated with mTORC1 and/or RSK inhibitors before MAPK pathway stimulation with PMA. Phosphorylation of immunoprecipitated HA-PDCD4 was analyzed with phospho-(S/T) Akt substrate antibody that preferentially recognizes phosphorylation on RXRXXpS/T. Based on RSK consensus phosphorylation site, this antibody may preferentially detect phosphorylation of PDCD4 at S67 and S457 [34]. Inhibition of RSKs reduced PDCD4 phosphorylation by ∼50% compared to vehicle-treated cells, while mTORC1 inhibition showed a modest effect (∼25%); the combined treatment had an additive effect (∼75%) (Figure 5A and 5B). We next evaluated the half-life of endogenous PDCD4 protein using the above-described treatment conditions in the presence of cycloheximide. MEK1/2 or RSK inhibitors significantly increased the half-life of PDCD4 (4.9h or 4.5h, respectively) compared to vehicle-treated cells (2.8 h.). In contrast, mTORC1 inhibitor produced a modest increase as single agent (3.2 h), but had a more significant impact on PDCD4 half-life in combination with MEK1/2 or RSK inhibitors (6.7h or 8.3h, respectively) (Figure 5C and 5D). These results indicated that phosphorylation of PDCD4 by RSKs promoted PDCD4 degradation in MDA-MB-231 cells. However, the phosphorylation of PDCD4 by mTORC1/S6K only showed a significant effect on protein stability when RSK activity was inhibited. The latter result suggested that mTORC1 may regulate PDCD4 expression by other mechanisms, since total PDCD4 levels were similarly elevated in the cells treated with mTORC1 or MAPK/RSK inhibitors (Figure 4C).

**Figure 6:**
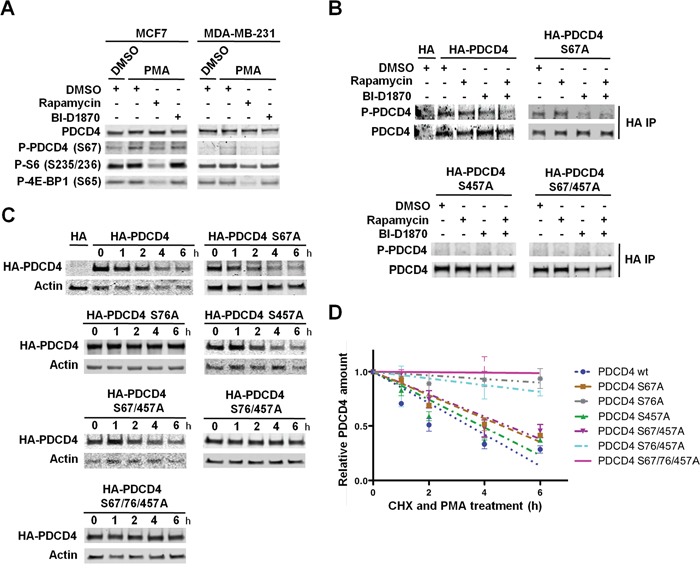
RSK-mediated phosphorylation of PDCD4 at S76 controls PDCD4 protein stability **A.** MCF7 and MDA-MB-231 cells were serum-starved for 24 h, then treated with vehicle (DMSO), rapamycin (20 nM), and/or BI-D1870 (10 μM) for 1 h, followed by stimulation with PMA (50 ng/ml) for 15 min. Indicated proteins were analyzed by immunoblots. **B.** MDA-MB-231 cells were transfected with an empty vector (HA) or constructs expressing HA-tagged PDCD4, PDCD4 (S67A), PDCD4 (S457A), or PDCD4 (S67/457A). Cells were serum-starved for 24 h, then treated with vehicle (DMSO), rapamycin (20 nM), and/or BI-D1870 (10 μM) for 1 h, followed by stimulation with PMA (50 ng/ml) for 30 min. HA-tagged PDCD4 proteins were immunoprecipitated with anti-HA antibody, and phosphorylation was analyzed with an anti-RXRXXpS/T motif antibody. **C.** MDA-MB-231 cells were transfected with an empty vector (HA) or constructs expressing HA-tagged PDCD4, PDCD4 (S67A), HA-PDCD4 (S76A), PDCD4 (S457), PDCD4 (S67/457A), PDCD4 (S76/457A), or PDCD4 (S67/76/457A). Transfected MDA-MB-231 cells were serum-starved for 24 h and then treated with cycloheximide (CHX; 100 μg/mL) and PMA (50 ng/ml). Cell extracts were prepared at the indicated time points, resolved by SDS-PAGE, and HA-tagged PDCD4 and actin proteins analyzed by immunoblotting. **D.** HA-tagged PDCD4 and actin levels were quantified using Image J software in immunoblots from three independent experiments performed as shown in C. HA-tagged PDCD4 values were normalized by actin values. Relative HA-PDCD4 values to respective t=0 controls (set to 1) were represented as means ± SEM, and the best-fit linear regression curves were calculated using Graphpad Prism 6.

We next identified the sites within PDCD4 that are phosphorylated by RSKs in PMA-stimulated MDA-MB-231 cells. First, we analyzed PDCD4 phosphorylation at S67 using a phospho-PDCD4 S67 antibody, since this site matches the RSK consensus phosphorylation motif [34]. We observed a modest increase of PDCD4 phosphorylation at S67, which was prevented by mTORC1 inhibition, but not by RSK inhibition (Figure 6A). Accordingly, we found a significantly increased phosphorylation at this site in MCF7 cells, in which PDCD4 levels were specifically regulated by PI3K/Akt/mTORC1 pathway (Figure 4A and 6A). The analysis of PDCD4 protein half-life showed that mutation of S67 to alanine, similar to mTORC1 inhibition, produced a modest increase, which indicated that mTORC1/S6K1-mediated phosphorylation at S67 was irrelevant for PDCD4 regulation in MDA-MB-231 cells (Figure 5C, 5D, 6C, and 6D). We next investigated the phosphorylation of PDCD4 at S457. To this end, HA-tagged PDCD4 (wt), PDCD4 (S67A), PDCD4 (S457A), or PDCD4 (S67/457A) proteins were expressed in MDA-MB-231 cells. Immunoprecipitated PDCD4 proteins were analyzed for PMA-induced phosphorylation with the phospho-(S/T) Akt substrate antibody described above. We observed that RSK inhibition reduced HA-PDCD4 phosphorylation and prevented the phosphorylation of HA-PDCD4 (S67A) mutant protein. Additionally, we did not detect phosphorylation of PDCD4 (S457A) mutant protein (Figure 6B). These results confirmed the phosphorylation of PDCD4 at S457 by RSKs. However, mutation of S457 did not result in increased half-life of PDCD4 compared to wild-type PDCD4 (3.9h and 3.5h, respectively) (Figure 6C and 6D). In addition to S457, S76 was also identified as a RSK phosphorylation site in melanoma cells [34]. This site maps within a canonical βTRCP-binding motif [D^70^SGRGD**S**^76^], and its phosphorylation is required for the binding of PDCD4 to βTRCP E3 ubiquitin ligase and consequently, for PDCD4 degradation [23, 34]. Unfortunately, we could not determine the phosphorylation of PDCD4 at S76 because there was not a commercially available phospho-PDCD4 S76 antibody, and the phospho-(S/T) Akt substrate antibody preferentially recognized phosphorylation at S67 and S457, as shown in Figure 6B. However, we found that mutation of S76 significantly increased PDCD4 half-life compared to S67A or S457A mutations (30h, 4.6h, and 3.9h, respectively). The triple mutant PDCD4 protein had a slightly increased half-life compared to PDCD4 S76A protein (Figure 6C and 6D). Taken together, these results indicate that phosphorylation of PDCD4 at S76 by RSKs is essential for the regulation of PDCD4 half-life in PMA-stimulated MDA-MB-231 cells.

### RSK-mediated down-regulation of PDCD4 promotes the proliferation, survival, and migration of MDA-MB-231 cells

**Figure 7:**
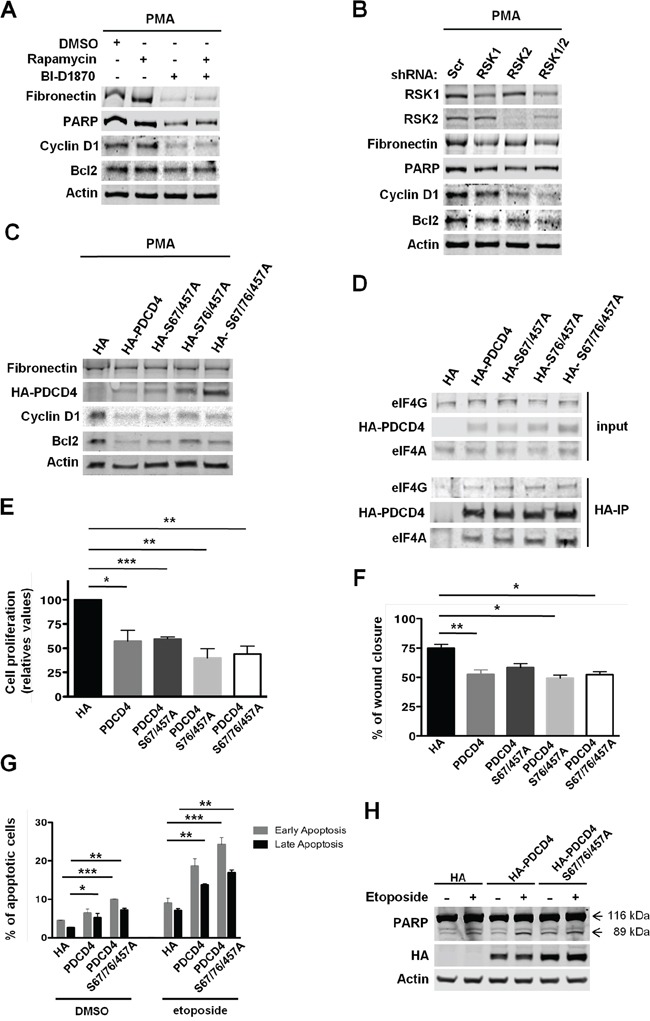
RSK-mediated regulation of PDCD4 is required for the proliferation, survival, and migration of MDA-MB-231 cells **A.** MDA-MB-231 cells were grown in serum-free media with PMA (50 ng/ml) and vehicle (DMSO), rapamycin (20 nM), and/or BI-D1870 (10 μM) for 24 h. Whole-cell extracts were obtained and resolved by SDS-PAGE. Indicated proteins were analyzed by immunoblotting with specific antibodies. **B.** MDA-MB-231 cells were infected with lentiviruses expressing shRNAs targeted against a scrambled sequence (Scr), RSK1, RSK2, or RSK1/2. After selection, cells were grown in serum-free media with PMA (50 ng/ml) for 24 h. Cell extracts were resolved by SDS-PAGE, and indicated proteins were analyzed by immunoblotting with specific antibodies. **C.** MDA-MB-231 cells transiently expressing HA tag, HA-tagged PDCD4, HA-tagged PDCD4 (S67/457A), HA-tagged PDCD4 (S76/457A), or HA-tagged PDCD4 (S67/76/457A) were selected and then grown in serum-free media with PMA (50 ng/ml) for 24 h. Indicated proteins were analyzed by immunoblotting with specific antibodies. **D.** Whole-cell extracts were obtained from the cells described in C. Equal amounts of total proteins were used to immunoprecipitate HA-tagged PDCD4 proteins using anti-HA agarose beads. Immunocomplexes and 1/10 of the protein used for immunoprecipitation (input) were resolved by SDS-PAGE, and indicated proteins were analyzed by immunoblotting with specific antibodies. **E.** MDA-MB-231 cells described in C were grown in 0.5% FBS media with PMA (50 ng/ml) for 3 days. Viable cells were estimated by neutral red uptake assays, and values represented as mean percentage ± SEM relative to HA tag-expressing cells (100%) determined from three independent assays (*p<0.05; **p<0.01; ***p< 0.001). **F.** Cells described in C were subjected to wound healing assays in 1% FBS media. Percentage of wound recovery was determined as described in Materials and Methods, and results were represented as means ± SEM. (*p<0.05; **p<0.01; ***p<0.001). **G.** Cells expressing HA tag, HA-tagged PDCD4, or HA-tagged PDCD4 (S67/76/457A) were selected and then treated with either vehicle (DMSO) or etoposide (50μM) for 24 hours. Early and late apoptotic cells were labeled with Guava Nexin Reagent, and quantified using the Guava EasyCyte Flow Cytometer. Percentage of apoptotic cells was represented as means ± SEM. (*p<0.05; **p<0.01; ***p<0.001). **H.** The levels of full-length (116 kDa) and cleaved (89 kDa) PARP, HA-PDCD4, and HA-PDCD4 (S67/76/457A) in cells treated as in G were determined by immunoblotting.

Tumor suppressor PDCD4 interacts with eIF4A, and preferentially inhibits the translation of mRNAs with highly structured 5′ UTRs [23, 24]. Thus, inhibition of eIF4A with silvestrol in MDA-MB-231 cells alters the translation of mRNAs with this structural complexity at the 5′ UTR that encodes factors involved in cellular proliferation, survival, and migration [37]. These data suggested that RSKs might translationally control the expression of these factors via the regulation of PDCD4. To evaluate this hypothesis, the levels of “eIF4A sensitive” factors such as cell cycle regulator Cyclin D1 and anti-apoptotic factor Bcl2 were determined in PMA-stimulated MDA-MB-231 cells. Unlike mTORC1 inhibition, the inhibition of RSKs reduced the levels of Cyclin D1 and Bcl2, and concomitantly induced apoptosis as determined by the decreased levels of full-length PARP (Figure 7A). Additionally, we observed reduced levels of Fibronectin, whose expression is regulated by RSKs at transcriptional level (Figure 7A) [38]. These results were further confirmed in RSK1/2-silenced cells (Figure 7B). In agreement with our hypothesis, over-expression of PDCD4 at different levels [HA-tagged PDCD4, PDCD4 (S67/457A), PDCD4 (S76/457), or PDCD4 (S67/76/457)] also inhibited the translation of *Cyclin D1* and *Bcl2* mRNAs (Figure 7C). As expected, Fibronectin did not change upon expression of PDCD4 proteins (Figure 7C). Additionally, we confirmed the inhibitory interaction of PDCD4 proteins with eIF4A and eIF4G by immunoprecipitation assays (Figure 7D). These results indicate that RSK-mediated down-regulation of PDCD4 is necessary for the translation of “eIF4A sensitive” mRNAs encoding factors involved in the proliferation, survival, and migration of TNBC MDA-MB-231 cells. Consequently, the over-expression of these PDCD4 proteins decreased the proliferation and migration of these cells, an effect similar to RSK inhibition or silencing, and increased their sensitivity to apoptosis induced by etoposide, as determined by the higher percentage of early and late apoptotic cells and elevated levels of cleaved PARP (Figure 1B, 2D, 2F, 7E, 7F, 7G, and 7H).

All together, these results indicate that RSK-mediated down-regulation of PDCD4 facilitates the translation of mRNAs encoding factors involved in cell cycle progression and survival, and therefore promotes proliferation, survival and migration of TNBC MDA-MB-231 cells.

## DISCUSSION

TNBC is a heterogeneous group of tumors that accounts for 15-20% of newly diagnosed breast cancer cases. These tumors respond to conventional chemotherapy but have a significantly higher probability of relapse and poorer overall survival in the first few years after diagnosis compared with other breast cancer subtypes. Unlike other breast cancer subtypes, targeted therapies for TNBC are not clinically available [39]. For this reason, it is critical to identify molecular drivers of these tumors that could be therapeutically targeted. High levels of RSK1 and/or RSK2 are detected in breast cancer tissues, particularly from TNBC patients, compared with normal tissues [5–7]. RSK inhibition or silencing of RSK1 and/or RSK2 reduce cellular proliferation, survival, migration, invasion, cancer stem cell growth, and tumor growth, preferentially in TNBC cell lines [6–13]. Thus RSKs have been proposed as putative targets for TNBC treatment. Interestingly, inhibition of RSKs does not affect the proliferation of normal breast epithelial cells, which suggests that therapeutic RSK inhibition may not produce the adverse side effects associated with MEK inhibitors [6, 40]. Herein, we show that the proliferation and migration of TNBC MDA-MB-231 cells, which harbor *KRAS* and *BRAF* mutations, selectively rely on RSK activity in response to PMA stimulation, but not on the activity of the PI3K/Akt/mTORC1 pathway. However, ER/PR-positive MCF7 cells, harboring an activating mutation in the *PI3KCA* gene, depend on both mTORC1 and RSK activities under the same conditions. These results confirm the critical role of RSKs in the control of TNBC cell growth, specifically of the cells with hyperactivated MAPK/RSK pathway [7, 8].

Increased protein synthesis is observed in many cancers, including breast cancer, and frequently arises as a consequence of elevated eIF4F activity. Deregulation of eIF4F activity results in increased translation of mRNAs that code for proteins involved in cellular growth and proliferation, survival, and migration, and consequently contributes to tumor development and progression [28, 41]. Accordingly, our data indicate that RSKs control proliferation and survival of MDA-MB-231 cells by regulating eIF4F activity. Unlike melanoma cells, this regulatory mechanism does not involve mTORC1 activity [25]. Specifically, RSKs control the activity of eIF4A, one of the components of eIF4F complex, through phosphorylation of eIF4B and PDCD4 in TNBC cells with up-regulated MAPK pathway. Phosphorylated eIF4B interacts with eIF4F, which results in increased ATPase and helicase activities of eIF4A [42–44]. Additionally, phosphorylation of PDCD4 promotes eIF4A activity by inducing PDCD4 degradation, and therefore, preventing the inhibitory interaction of PDCD4 with eIF4A [23, 24]. RSK-induced degradation of PDCD4 may also prevent the inhibitory activity of PDCD4 on the translation elongation of specific mRNAs [45]. Interestingly, the regulation of eIF4B and PDCD4 is mediated by specific RSK isoforms. Our results suggest that RSK3 and/or RSK4 may phosphorylate eIF4B, since phosphorylation is not reduced in RSK1/2-silenced MDA-MB-231 cells. Consistently, over-expression of RSK3 or RSK4 partially prevents dephosphorylation of eIF4B in MCF7 cells treated with PI3K inhibitors [5]. In contrast, we show that RSK1/2 phosphorylate and consequently promote the degradation of PDCD4 in MDA-MB-231 cells. Similar results have been recently reported in melanoma cells [34]. Specifically, we have found that the phosphorylation of PDCD4 at S76 induces protein degradation, probably by facilitating the 14-3-3-mediated binding of PDCD4 to the β-TRCP E3 ubiquitin ligase, as previously reported [23, 34]. PDCD4 S457 also participates in this regulatory mechanism in melanoma cells but, although phosphorylated, this site does not show a significant effect on PDCD4 degradation in MDA-MB-231 cells. In addition, we observed that mTORC1-dependent phosphorylation of PDCD4 at S67 slightly contributes to protein degradation, although its effect becomes more significant when the MAPK/RSK pathway is inhibited.

In agreement with RSK-mediated regulation of eIF4A activity in MDA-MB-231 cells, we observed that the translation of “eIF4A sensitive” mRNAs such as *Cyclin D1* and *Bcl2* requires RSK activity and, concomitantly, low levels of PDCD4. These results are consistent with the requirement for RSK activity or low PDCD4 levels for the proliferation, survival, and migration of these cells. Additionally, they suggest that this regulatory mechanism plays an important role in the development and progression of TNBC in which MAPK pathway is up-regulated. Interestingly, we detected much lower levels of PDCD4 in TNBC cell lines with hyperactivated MAPK pathway than in the other breast cancer cell lines used in this study. These TNBC cell lines show a mesenchymal-like phenotype, which has been associated with low PDCD4 levels [46, 47]. Accordingly, low levels of PDCD4 promote metastasis, and are associated with more aggressive breast cancers [47–49]. Moreover, high levels of eIF4A are independent predictors of poor outcome in ER-negative breast cancer [50].

These results indicate that RSK-mediated translational control is essential for proliferation, survival, and migration of MDA-MB-231 cells, and therefore may contribute to the development and progression of TNBC subtypes with up-regulated MAPK/RSK pathway. These results further contribute to the emerging interest in RSKs and/or PDCD4 as promising therapeutic targets for the treatment of TNBC and other cancers with up-regulated MAPK pathway such as lung, colorectal, pancreatic and melanoma. Unfortunately, although RSK inhibitors have been developed and shown promising results in preclinical studies, there are yet no clinically available inhibitors [3, 6, 11, 51, 52]. The development of more selective and isoform-specific RSK inhibitors might significantly contribute to better understanding of the role of RSKs in tumorigenesis in preclinical models and serve as a basis for new therapeutic approaches for treatment of TNBC.

## MATERIALS AND METHODS

### Cell culture and reagents

HEK-293T, MCF7, MDA-MB-231, BT474, T47D, and MDA-MB-468 were maintained in Dulbecco's modified Eagle medium (DMEM; Corning, Manassas, VA, USA), supplemented with 10% fetal bovine serum (FBS; Corning), and Penicillin/Streptomycin (Corning). SUM159PT cells were maintained in Ham's medium (Corning) supplemented with 5% FBS, Hepes (10mM; Sigma-Aldrich, St. Louis, MO, USA), Insulin (5 μg/ml; Sigma-Aldrich), Hydrocortisone (1 μg/ml; Sigma-Aldrich), and Penicillin/Streptomycin. MDA-MB-436 cells were maintained in DMEM:Ham's (1:1) supplemented with 10% FBS, L-Glutamine (2mM; HyClone, Logan, UT, USA), and Penicillin/Streptomycin. Dimethyl sulfoxide (DMSO; Sigma-Aldrich), rapamycin (20 nM; Sigma-Aldrich), U0126 (10 μM; Cell Signaling Technology, Danvers, MA, USA), BI-D1870 (10 μM; Santa Cruz Biotechnology, Dallas, TX, USA), insulin (100 nM; Sigma-Aldrich), phorbol 12-myristate 13-acetate (PMA) (50 ng/ml; Sigma-Aldrich), etoposide (50 μM; Sigma-Aldrich), and cycloheximide (100 μg/ml; Sigma-Aldrich) were used as indicated in figure legends.

### Plasmids and transfections

pcDNA3-HA was kindly provided by N. Sonenberg [53]. Human wild-type PDCD4 was cloned in frame with a triple HA sequence into pKH3 vector to generate pKH3-HA-PDCD4. pKH3-HA-PDCD4 (S67A), pKH3-HA-PDCD4 (S76A), pKH3-HA-PDCD4 (S457A), pKH3-HA-PDCD4 (S67/457A), pKH3-HA-PDCD4 (S76/457A), and pKH3-HA-PDCD4 (S67/76/457A) plasmids were constructed by overlap extension PCR mutagenesis using plasmid pKH3-HA-PDCD4 as template.

MDA-MB-231 cells were transfected using Lipofectamine 3000 (Invitrogen, Grand Island, NY, USA) according to the manufacturer's protocol. To select PDCD4 over-expressing MDA-MB-231 cells, cells were co-transfected with PDCD4 plasmids and pcDNA3-HA vector (harboring neomycin resistance gene), and selected in media containing geneticin (500 μg/ml; G418 disulfate salt; Sigma-Aldrich).

### Lentiviral transduction and gene silencing

Plasmids GIPZ-NS Control (RHS 4346; Dharmacon, Lafayette, CO, USA), GIPZ-RPS6KA1 (RHS 4430-98901284; Dharmacon), and GIPZ-RPS6KA3 (RHS 4430-99297973; Dharmacon) were purchased from the Albert Einstein College of Medicine shRNA Core Facility.

Viral production was carried out by co-transfecting viral plasmids and plasmids expressing Rev, Tat, Gag/Pol, and VSV-G into HEK-293T cells. Virus-containing media were collected at 48 h and 72 h post-transfection, centrifuged at 2500 rpm for 10 min at 4°C, and filtered (0.45 μM). MDA-MB-231 cells were infected with virus-containing media in the presence of 5 μg/ml Polybrene (Sigma-Aldrich) for 24 h. Infected cells were selected in media containing puromycin (2 μg/ml; Sigma-Aldrich).

### Immunoblot analysis and immunoprecipitation assays

For immunoblot analysis, cell lysates were prepared by incubating the cells in RIPA buffer (150 mM NaCl, 10 mM Tris pH7.4, 0.1% SDS, 1% Sodium Deoxycholate, 5 mM EDTA, 1% Triton X-100, 50 mM NaF, 25 mM β-Glycerophosphate, 40 μg/ml PMSF, 10 μg/ml Leupeptine, 5 μg/ml Pepstatin, and 10 μg/ml Aprotinin) for 20 min on ice, followed by centrifugation at 10k rpm at 4°C for 10 min. Equal amounts of whole-cell extracts were resolved by SDS-PAGE (4-12% gradient; Invitrogen), and transferred to nitrocellulose membrane (GE Healthcare, Pittsburg, PA, USA). Indicated proteins were detected by immunoblot analysis using specific antibodies: anti-eIF4B, anti-phospho-eIF4B (Ser422), anti-PDCD4, anti-phospho-S6 (S235/236), anti-4EBP1, anti-phospho-4EBP1 (S65), anti-phospho-(S/T) Akt substrate, anti-Cyclin D1, anti-Bcl2, anti-PARP, anti-eIF4G, anti-eIF4A, anti-RSK2 (S227) (Cell Signaling Technology); anti-phospho-ERK and anti-Fibronectin (Sigma-Aldrich); anti-actin, and anti-RSK2 (Santa Cruz Biotechnology); anti-PDCD4 (S67) (Millipore, Billerica, MA, USA); anti-RSK1 and anti-HA 12CA5 (Abcam, Cambridge, UK). Anti-goat IRDye, anti-mouse IRDye, and anti-rabbit IRDye (LI-COR Biosciences, Lincoln, NE, USA) were used as secondary antibodies.

For immunoprecipitation assays, cells were lysed using lysis buffer (10 mM K_3_PO_4_, 1 mM EDTA, 10 mM MgCl_2_, 5 mM EGTA, 50 mM β-Glycerophosphate, 50 mM NaF, 0.5% Nonidet P-40, 0.1% Brij, 40 μg/ml PMSF, 10 μg/ml Leupeptin, 5 μg/ml Pepstatin, and 10 μg/ml Aprotinin). Equal amounts of protein were incubated with anti-HA agarose (Sigma-Aldrich) overnight at 4°C. Beads were washed five times with lysis buffer, and complexes were resolved and detected as described above.

### Cell proliferation and foci formation assays

For foci formation, MDA-MB-231 cells were seeded in duplicate into 6-well plates at 500 cells/well. Cells were cultured in indicated media for 14 d, replacing the media every 3 days. Colonies were fixed with -20°C pre-cooled methanol for 10 min and stained with 0.5% crystal violet in 25% methanol for 10 min. Colonies were washed with water. Only foci of 50 or more cells were scored.

Cell viability was determined by neutral red uptake assays as described in [31]. Briefly, MCF7 or MDA-MB-231 cells were seeded in duplicate into 96-well plates at 5,000 cells/well. Cells were cultured in indicated media for 3 days. Cells were incubated in 100 μl of 0.5% FBS DMEM containing Neutral Red (40 μg/ml; Sigma-Aldrich) for 2 h, washed twice with 150 μl of 1xPBS, and destained for 10 min with 50% ethanol: 49% H_2_O: 1% acetic acid. Absorbance was measured at 540 nm.

### *In vitro* wound healing assay

MCF7 or MDA-MB-231 cells were seeded into 6-well plates and incubated in 10% FBS DMEM until confluent monolayer was formed. Scratches were generated with sterilized p20 pipette tips. Cells were washed twice with 1xPBS to remove unattached cells. Cells were incubated as described in the figure legends. Images were captured using an inverted phase-contrast microscope (EVOS^®^ FL Auto Cell Imaging System; ThermoFisher Scientific, Waltham, MA, USA) at the indicated times. Wound size was measured using ImageJ software (NIH Image), and percentage of wound closure was determined.

### Apoptosis assays

MDA-MB-231 cells expressing HA, HA-PDCD4, or HA-PDCD4 (S67/76/457A) were treated with vehicle (DMSO) or etoposide (50 μM) for 24 h. The cells were labeled with Guava Nexin Reagent (Guava Technologies, Hayward, CA, USA) according to the manufacturer's protocol. Cells were sorted using a Guava EasyCyte Flow Cytometer (Guava Technology, Millipore) and analyzed with GuavaSoft software (Guava Technology).

### Statistical analysis

Statistical analysis was performed using the Prism Graphpad 6 software. Significance was determined by paired two-tailed Student's t-test. P-values < 0.05 were considered significant.
